# The Relationship between Job Satisfaction and Depressive Symptoms among Chinese Adults Aged 35–60 Years: The Mediating Role of Subjective Well-Being and Life Satisfaction

**DOI:** 10.3390/ijerph20032023

**Published:** 2023-01-22

**Authors:** Yixuan Liu, Xinyan Yang, Yinghui Wu, Yanling Xu, Yiwei Zhong, Shujuan Yang

**Affiliations:** Department of Social Medicine and Health Management, School of Public Health, Jilin University, Changchun 130021, China

**Keywords:** job satisfaction, depressive symptoms, subjective well-being, life satisfaction

## Abstract

The purpose of this study was to assess the serial multiple mediating effects of subjective well-being and life satisfaction between job satisfaction and depressive symptoms among Chinese adults aged 35–60 years. According to the 2018 China Family Panel Study (CFPS), we finally selected 10,609 respondents (5202 females, and 5407 males) aged 35–60 years old as samples for the study. Correlation analysis was carried out to examine the relationship among job satisfaction, subjective well-being, life satisfaction, and depressive symptoms. Linear regression models were established to analyze the relationship between job satisfaction and depressive symptoms. Serial multiple mediation analysis was conducted by the SPSS macro PROCESS program. The results suggested that job satisfaction was negatively correlated with depressive symptoms among Chinese adults aged 35–60 years. Subjective well-being and life satisfaction mediated the relationships between them, respectively. Furthermore, job satisfaction also had indirect impacts on depressive symptoms through the serial mediating effects of subjective well-being and life satisfaction. The findings revealed that increasing job satisfaction could decrease depressive symptoms through promoting subjective well-being and life satisfaction. The study may offer some meaningful implications for improving the mental health and reducing the risk of depressive symptoms among Chinese adults aged 35–60 years.

## 1. Introduction

Depressive disorder is one of the most common mental disorders. It refers to a kind of mood disorder caused by various reasons and is characterized by significant and persistent depression. With the development of the economy, the pace of people’s life has become faster and faster, and the pressures of life are becoming larger and larger, and both depressive symptoms including major depression are on the rise among the population. Depression has become a widespread and significant mental health problem worldwide and is a major contributor to the global burden of disease [1]. According to the World Health Organization, depression ranked third in the global burden of disease in 2008 and is expected to rank first in the world by 2030. Depression is a widespread and significant mental health problem worldwide. If this trend is not halted, the burden of depression is expected to continue to grow including harmful effects on people’s quality of life and excessive mortality rates [2,3]. In recent research, the prevalence of depression among people was reported to be high. A cross-sectional study in the United States showed that from 2005 to 2015, the prevalence of depression among people aged 12 and above increased significantly, especially among youth groups compared to older groups [4]. A German data survey covering 87 percent of the population found that the prevalence of depression increased from 12.5 percent in 2009 to 15.7 percent in 2017 [5]. A sampled population of one million individuals in Korea reported that the prevalence of depression increased from 2.8% in 2002 to 5.3% in 2013 [6]. A national survey in China showed that approximately 30% of men and 43% of women aged 45 years and older experienced depressive symptoms [7]. A survey of 3056 Chinese teenagers aged 10–15 showed a 16.7% prevalence of depression [8]. It is important to note that the majority of studies concerning depression have focused on adolescents and/or the elderly, while studies on the middle-aged are relatively understudied. According to Erikson’s stages of psychosocial development, compared with other ages, people in the middle-aged phase (35–60 years old) are experiencing the changes of all kinds of sense organs including the gradual deterioration of some physical functions. This stage is named the generativity versus stagnation stage of development [9]. At the same time, adults in this age group are at a stage where their career development, family cycles, and life cycles overlap. They also face pressures from working, supporting parents, raising children as well as managing and maintaining marriages and relationships [10]. All of these may lead to having some influence on the adults’ mental health, which manifests as unstable mental state and mood changes such as irritability or anxiety together with other negative emotions, and may even cause depression [11,12]. Previous studies have found that as a common chronic condition, depression is the leading cause of mortality in middle-aged adults [13]. Therefore, understanding the modifiable factors related to depressive symptoms in middle-aged adults is of great significance to ameliorate the incidence of depression and improve their mental health.

Many studies have shown that sociodemographic characteristics (gender [14], age [15], et al.), living styles and habits (diet [16], exercise habits [17], et al.), poor physiological health [18], and mental health [19] status are associated with the increased risk of depression. In addition, other factors such as social relationships (e.g., social trust, social support) [20] and negative life events [21] strongly correlate with the prevalence of depressive symptoms. Significantly, job satisfaction has also been proven to be a related factor for depressive symptoms [22]. Job satisfaction is a pleasurable or positive emotional state resulting from the appraisal of one’s job or job experiences [23], which is usually influenced by factors such as salary, work safety, environment, hours, and promotion [24]. As a job-specific attitude, job satisfaction should be considered as an important indicator of mental health in the workplace [25]. It is well-known that when people are not satisfied with their job, they will have negative emotions, which in serious cases can even lead to certain mental illnesses such as depression. A previous survey on health care workers in Brazil observed that higher job satisfaction was a protective factor for depression, which could reduce the frequency of poor mental health outcomes [26]. Similarly, a cross-sectional study carried out in Peru indicated that workers with low job satisfaction had a higher probability of developing mental health issues such as depressive symptoms [27]. A meta-analysis involving 43,884 rural-to-urban migrant workers in China showed that job satisfaction was correlated with depressive symptoms [28]. Another 1-year prospective study among Japanese civil servants demonstrated that job dissatisfaction could predict the development of depression after a short period [29]. A large study based on company and population found that the perception of negative psychosocial factors in the workplace including job dissatisfaction was associated with an increased risk of subsequent depressive symptoms [30]. At the same time, several studies have focused on the relationship between some aspects implicated with job satisfaction (such as job demands, stress) and depressive symptoms. For example, in a study of full-time employees at all levels in Canada, Melchior et al. [31] reported that those with high psychological job demands such as excessive workload and extreme time pressures had a twofold risk of developing major depression when compared to individuals with low demands. Linking job stress to depression, Wang and Patten hypothesized that employees who reported high psychological demands, low decision latitude, high levels of physical exertion, job insecurity, or lack of support from co-workers would be more likely to suffer from major depression than individuals who reported low stress in those areas [32]. Combing through the studies on the relationship between job satisfaction and depression, we found that most of the studies were based on a specific occupation (doctors [27] or nurses [33]), limited sample areas (data were usually collected in a specific workplace [34]), or limited sample size (less than 10,000 respondents [29]), so the conclusions acquired were targeted and difficult to generalize. As the main workers in current society, adults aged 35–60 years usually carry the responsibility and burden of working, so the association between their job satisfaction and depressive symptoms is still worth further exploration. Therefore, we are committed to exploring the relationship between job satisfaction and depressive symptoms and its underlying mechanisms in Chinese adults aged 35–60 years through nationally representative data. The first hypothesis we propose is that there is some relationship between job satisfaction and depressive symptoms in Chinese adults aged 35–60 years.

In the current research on the correlation between psychological factors and depressive symptoms, most studies have focused on negative emotions and ignored the impact of positive emotions that are primarily concerned with positive aspects of life such as subjective well-being and life satisfaction [35]. Subjective well-being (SWB) is an individual’s emotional evaluation for the degree and content of positive emotional experiences at happy moments in their life, which tends to be an affective component [36]. A large number of empirical studies have found that job satisfaction has a positive effect on the subjective well-being of people. A study of 971 Portuguese-speaking adults indicated that the existence of the protector effect of job satisfaction for health, happiness, and subjective well-being [37]. Xu and Xia analyzed the trend of the evaluation of the Chinese residents’ subjective well-being and its influencing factors over the past two decades and found that people who were satisfied with their jobs hade higher subjective well-being levels [38]. By analyzing the influence of specific factors related to the work domain on the employees’ subjective well-being, Erdogan et al. found that job satisfaction had the most significant effects [39]. Although there have been few studies on the influence of subjective well-being on depression, previous studies have demonstrated that the increase in subjective well-being can effectively solve people’s mental health problems [40], which have a close connection with less depressive symptoms. By establishing a BP neural network model, Fan et al. found that subjective well-being was the most significant factor affecting depressive symptoms in Chinese middle-aged and elderly people [41]. A cross-sectional study involving 14,344 adults indicated that higher levels of subjective well-being were related to a lower risk of depression [42]. Given all of this, it is of great significance to explore the role of subjective well-being related to positive emotions in the potential relationship between job satisfaction and depressive symptoms. We proposed a second hypothesis that subjective well-being might mediate the association between job satisfaction and depressive symptoms.

As another key indicator of the assessment for positive emotions and a reflection of the good life, life satisfaction can be defined as the individuals’ perceptual evaluation of their own life quality according to a set of specific criteria [43]. Unlike subjective well-being, which emphasizes a spontaneous transient experience, life satisfaction focuses on the long-term sense of achieving lifelong goals and mainly contains a cognitive component [36]. Although the definition or evaluation of life satisfaction does not necessarily include the meaning and satisfaction of a job, higher job satisfaction can be important for building overall life satisfaction [44]. Different studies have reported some positive and significant correlations between satisfaction in their job and life. A study about Chinese university teachers revealed a positive relationship between job satisfaction and life satisfaction [45]. In the U.S., Liu et al. [46] concluded that job satisfaction is one of the main domains that explain life satisfaction. A cross-sectional study that included 559 civil servants aged 27 to 60 years old in China reported that increasing job satisfaction might be important measures to improve their life satisfaction [47]. In terms of the research on the relationship between life satisfaction and depressive symptoms, multiple studies have confirmed that life satisfaction is negatively related to the occurrence of depression, with lower life satisfaction related to the increase in depressive symptoms. A large population-based cohort study previously demonstrated that poor life satisfaction was associated with increased development of multiple chronic diseases such as depression and a higher risk of death [48]. A study on Turkish undergraduate students found that life satisfaction was negatively correlated with negative emotions [49], which may, in turn, lead to depression. Mei et al. found that people with poor life satisfaction were more likely to have a high level of depression and anxiety [50]. Although subjective well-being and life satisfaction are broad rather than specific perceptions, they are important contributors to positive emotional experience and have been widely studied because of their interactive effects on mental health [51,52]. Actually, as an emotional cognition-judgment component of one’s life, subjective well-being is positively correlated with life satisfaction [53]. A study on the subjective well-being of youth in China found that personal subjective happiness was closely related to life satisfaction [54]. Using panel data from Germany, researchers found that how happy people thought had a greater impact on their life satisfaction than their actual circumstances [55]. In the study of subjective well-being, it is equally important to examine life satisfaction [54]. Studies have shown that older adults who spontaneously experience subjective well-being and have higher life satisfaction report less depressive feelings [56]. According to the empirical evidence about the relationship between job satisfaction, subjective well-being, life satisfaction, and depressive symptoms, we proposed the third and fourth hypotheses that life satisfaction might be a potential mediator between job satisfaction and depressive symptoms and that subjective well-being and life satisfaction would have sequential mediating effects between job satisfaction and depressive symptoms, respectively.

In conclusion, based on nationally representative data, the aim of this study was to: (1) explore the relationship between job satisfaction and depressive symptoms in Chinese adults aged 35–60 years and (2) examine the roles of subjective well-being and life satisfaction on the association between job satisfaction and depressive symptoms from the perspective of positive emotions. This study will complement and enrich the current knowledge about the factors related to depressive symptoms and its internal influence mechanism in middle-aged people. Moreover, these findings could provide a valuable reference for the development of interventions aimed at preventing depressive symptoms and promoting mental health in middle-aged adults.

## 2. Materials and Methods

### 2.1. Data Source and Sample

The data were from the 2018 wave of the China Family Panel Studies (CFPS), which is funded by the 985 Program of Peking University. CFPS is implemented by the Chinese Center for Social Science Surveys (ISSS) at Peking University. It aims to reflect the changes in China’s society, economy, population, education, and health by tracking and collecting data at individual, family, and community levels, and provide a data basis for academic research and public policy analysis. CFPS focuses on the economic and non-economic well-being of Chinese residents as well as a wide range of research topics including economic activity, educational outcomes, family relationships and dynamics, population migration, and health. CFPS is a national, large-scale, multidisciplinary social tracking survey. The CFPS sample covers 25 provinces/municipalities/autonomous regions with a target sample size of 16,000 households, and all members of the sample households are included in the survey. CFPS conducted preliminary and follow-up test surveys in Beijing, Shanghai, and Guangdong, respectively, in 2008 and 2009, and officially launched the survey in 2010. All baseline family members identified by the 2010 baseline survey and their future biological/adopted children will be permanently tracked as CFPS genetic members. CFPS has four main questionnaire types: community questionnaire, family questionnaire, adult questionnaire, and children questionnaire. On this basis, CFPS has developed a variety of questionnaire types such as long questionnaire, short questionnaire, proxy questionnaire, and telephone interview questionnaire for different family members.

In the 2018 CFPS samples, the survey was based on the households defined in the 2010–2016 national survey and collected 32,669 valid individual samples. Due to research needs, we selected adults aged 35–60 years old as samples for the study. After core variables containing missing values had been deleted, our final analytical samples consisted of 10,609 respondents.

### 2.2. Measurements

#### 2.2.1. Depressive Symptoms

Depression symptoms were measured by the eight-item short version of the Center for Epidemiologic Studies Depression Scale (CES-D8) [57]. In this study, the brief self-report 8-item version of CES-D was applied, which were six positive questions and two negative questions. CES-D-8 was used to measure the emotion frequency in the previous week as follows: (1) I felt depressed; (2) I felt that everything I did was an effort; (3) My sleep was restless; (4) I felt happy; (5) I felt lonely; (6) I enjoyed life; (7) I felt sad; and (8) I could not get going. The item responses were assessed on a 4-point scale where 0 represents “rarely or never” (less than 1 day), 1 “not too often” (1–2 days), 2 “sometimes or half the time” (3–4 days), and 3 “most of the time” (5–7 days). The total scores ranged from 0 to 24. The higher the score, the greater level or intensity of depressive symptoms [58]. CES-D8 has been widely proven to have reliability and validity in measuring depressive symptoms [59,60,61]. The Cronbach’s alpha coefficient of depressive symptoms in this study was 0.766.

#### 2.2.2. Job Satisfaction

Referring to previous studies [62,63], job satisfaction was indicated by the degree of people’ satisfaction in the following six aspects: work income, work safety, work environment, work hours, work promotion, and general feelings about work. The answers ranged from 1 (very dissatisfied), 2 (dissatisfied), 3 (generally), 4 (satisfied), and 5 (very satisfied). The sum of the answers to all the questions can represent how satisfied people are with their jobs. Finally, the total score was on a scale of 6 to 30, which means that the higher the score, the more satisfied people are with their jobs. The Cronbach’s alpha coefficient of job satisfaction in this study was 0.743.

#### 2.2.3. Subjective Well-Being

It has been found that the method of directly collecting the data about the respondents’ subjective well-being is the most reliable, effective, and comparable [64,65]. Therefore, consistent with previous studies [66,67], subjective well-being was measured by a 5-point Likert-type scale question: “What is your happiness level?” answered ranging from “1 (very unhappy)”, “2 (unhappy)”, “3 (general)”, “4 (happy)”, “5 (very happy)”. The respondents rated on a five-point scale, in which higher scores indicate higher levels of subjective well-being.

#### 2.2.4. Life Satisfaction

Life satisfaction lays its foundation on the response of the resident for “In general, how satisfied are you with your present life?”. For all items, the answers represented a Likert-type format of 5 points, ranging from 1 (very dissatisfied) to 5 (very satisfied). This measurement method has also been verified to be representative and reliable by previous studies [68,69].

#### 2.2.5. Other Covariates

The covariates in this research included age, gender (0 = female, 1 = male), self-reported health (1 = very unhealthy, 2 = unhealthy, 3 = general, 4 = healthy, 5 = very healthy), and marital status (not married = 0, married = 1). “Not married” was coded into the following four categories, which were “single”, “cohabitation”, “divorced”, and “widow”.

### 2.3. Statistical Analysis

In this study, we used IBM SPSS Statistics version 24 to process the data. Descriptive statistics were conducted to describe the sociodemographic characteristics of the study population, and a correlation analysis was used to verify the relationship between the variables. Linear regression models were established to analyze the relationship between job satisfaction and depressive symptoms. Serial multiple mediation effects of subjective well-being and life satisfaction between job satisfaction and depression symptoms were estimated via the SPSS macro PROCESS program (Model 6) designed by Hayes [70]. The study set the bootstrap confidence interval (CI) at 95% based on 5000 bootstrapped samples. If zero was not included in the interval of 95% CI, it indicated that the mediation effect was significant.

## 3. Results

### 3.1. Characteristics of Samples

Table 1 shows the demographic characteristics of the participants. There were 10,609 respondents included in this study, and the mean age of the respondents was 47.91 ± 6.88 years old. Among them, 5202 (49.0%) were females, and 5407 (51.0%) were males. In terms of marital status, the majority of respondents were married (95.0%). From the perspective of the self-reported health conditions, 25.9% of respondents thought that they were in good health, 31% did not think that they were healthy.

### 3.2. Correlation of Job Satisfaction, Subjective Well-Being, Life Satisfaction, and Depressive Symptoms

The correlations between the primary relevant variables are shown in Table 2. Job satisfaction was positively correlated with subjective well-being (r = 0.144, *p* < 0.001) and life satisfaction (r = 0.228, *p* < 0.001). Subjective well-being was also positively correlated with life satisfaction (r = 0.398, *p* < 0.001). Depressive symptoms were significantly negatively correlated with job satisfaction, subjective well-being, and life satisfaction, with correlation coefficients of −0.109, −0.321, and −0.230, respectively. The results of the correlation analysis were consistent with our expected hypotheses, and all the analysis results were statistically significant at the level of *p* < 0.05 (two-tailed).

### 3.3. Mediation Analysis of Subjective Well-Being and Life Satisfaction

As shown in Table 3, Model 1 mainly explored the relationship between job satisfaction and depressive symptoms. The result showed that job satisfaction had a significant association with depressive symptoms (B = −0.134, *p* < 0.001). Model 2 reported the association between job satisfaction and depressive symptoms when adjusting for all the covariates. Although the coefficient reduced, job satisfaction was still significantly correlated with depressive symptoms (B = −0.0902, *p* < 0.001). In addition, age (B = −0.0117, *p* < 0.05) and self-reported health (B = −0.9671, *p* < 0.001) were associated with depressive symptoms. Respondents who were males (B = −0.8347, *p* < 0.001) or married (B = −1.7920, *p* < 0.001) had a lower risk of depressive symptoms. In conclusion, the results of Model 1 and Model 2 demonstrated that job satisfaction was an important predictor of depressive symptoms in Chinese adults aged 35–60 years.

**Figure 1:**
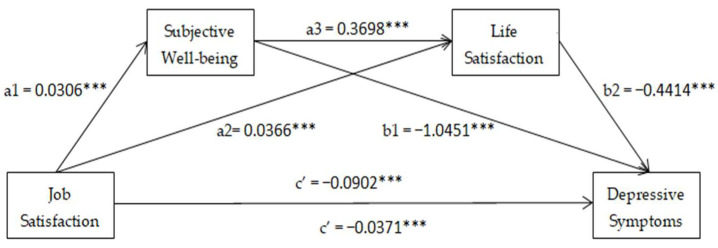
Serial mediation models for job satisfaction, subjective well-being, life satisfaction, and depressive symptoms. Note: Path coefficients were shown in standardized regression coefficient. *** *p* < 0.001.

In order to investigate the internal mechanism of the relationship between job satisfaction and depressive symptoms, we further examined the mediating effect of subjective well-being and life satisfaction. Figure 1 displays the coefficients and significance of the relationship among job satisfaction, depressive symptoms, subjective well-being, and life satisfaction after controlling for covariates. Job satisfaction had a significant and negative correlation with depressive symptoms (B = −0.092, 95%CI: −0.1058, −0.0746). Job satisfaction had a significant and positive association with subjective well-being (B = 0.0306, 95%CI: 0.0264, 0.0348). Subjective well-being had a significant and positive correlation with life satisfaction (B = 0.3698, 95%CI: 0.3483, 0.3905). Job satisfaction had a significant and positive association with life satisfaction (B = 0.0366, 95%CI: 0.0327, 0.0407). Life satisfaction had a significant and negative correlation with depressive symptoms (B = −0.4414, 95%CI: −0.5292, −0.3578). When controlling for subjective well-being and life satisfaction, job satisfaction was still negatively correlated with depressive symptoms, although the coefficient decreased (B = −0.0371, 95%CI: −0.0531, −0.0206). Table 4 shows the bootstrapping results for the mediation analysis, which meant that all the indirect and direct paths were significant. Specifically, the first indirect pathway was that subjective well-being significantly mediated the effect of job satisfaction on depressive symptoms, with an effect value of −0.032. The second indirect pathway was that the effect of job satisfaction on depression was significantly mediated by life satisfaction, with an effect value of −0.0162. The third indirect pathway was that the effect of job satisfaction on depressive symptoms was significantly mediated by both subjective well-being and life satisfaction, with an effect value of −0.005. Furthermore, the total and direct effect of job satisfaction on depressive symptoms was −0.0902 and −0.0371, respectively. In conclusion, these results suggest that subjective well-being and life satisfaction play an independent or serial mediating role in the relationship between job satisfaction and depressive symptoms.

## 4. Discussion

In this study, the main purpose was to explore the relationship between job satisfaction and depressive symptoms among Chinese adults aged 35–60 years and the underlying mechanisms behind them. The findings showed that job satisfaction had a significant direct influence on depressive symptoms. At the same time, subjective well-being and life satisfaction partially mediated the relationship between job satisfaction and depressive symptoms, respectively. Finally, subjective well-being and life satisfaction also had the combined mediating effects between job satisfaction and depressive symptoms. In consideration of the analysis results obtained, all of the hypotheses we made at the beginning of the study were supported.

### 4.1. The Direct Effect of Job Satisfaction on Depressive Symptoms

The results suggest that job satisfaction is negatively correlated with depressive symptoms among Chinese adults aged 35–60 years, which means that a lower level of job satisfaction indicates a higher risk of depressive symptoms. The findings of this study adhere to those in previous research [71,72]. Some meta-analyses have found that job satisfaction had a significant negative effect on depression [73,74], which suggests that higher job satisfaction results in less depression. Although job satisfaction is a concept based on the worker’s perception, it directly reflects the actual conditions of the workplace [27]. Poor working conditions are often associated with low income and low job security, the absence of pensions and health benefits, greater risk of layoff, and lack of social protection from unions and labor laws for employees [75,76,77,78]. For the majority of adults aged 35–60 years, the increasing pressure from family and life makes it difficult for them to maintain their enthusiasm for work and to even develop their complaints and dissatisfaction with the working conditions that are already ordinary. Anxiety and disappointment about the working conditions could cause emotional distress and to some extent induce the emergence and development of depressive symptoms [79]. There is substantial evidence to support the relationship between job dissatisfaction and depressive symptoms because the former predisposes one to negative emotional feelings [80]. If these negative feelings do not go away for a long time, or become so intense that one cannot control them, the person is likely to gradually suffer from depression. Therefore, it is crucial to ensure that adults aged 35–60 years are satisfied with the work itself as it contributes to limiting the sources of negative emotions and have better access to mental health care, all of which are protective factors for depressive symptoms.

### 4.2. The Mediation Effect of Subjective Well-Being and Life Satisfaction

The study also examined the potential mechanism between job satisfaction and depressive symptoms from the perspective of positive emotions, which revealed the mediating roles of subjective well-being and life satisfaction. The results indicate that the indirect effect between job satisfaction and depressive symptoms among Chinese adults aged 35–60 years can be mediated by subjective well-being and life satisfaction, respectively. Consistent with other research findings [81], higher job satisfaction contributes to the development of personal identity and enables people to participate in social communication with a healthy frame of mind [37]. In other words, the individuals’ positive evaluation of their actual work status makes them more likely to catch the gorgeous moments in life and increases their subjective well-being, which in turn positively influences mental performance and provides individuals with protection from depression [41]. Conversely, the period of middle adulthood is often vulnerable to the occurrence of adverse work changes and a lack of job satisfaction, resulting in feelings of lower subjective well-being, and, consequently, obvious depressive symptoms [82]. Like subjective well-being, job satisfaction has also been linked to a host of other positive variables such as greater life satisfaction [83]. Satisfaction with job-related evaluation indicators such as salary, hours, and promotion opportunities increases the individuals’ positive emotional experiences for other aspects of life, which gives them more confidence to tackle challenges in life and also acts as a protective factor to reduce negative emotional experiences, thus effectively preventing depression [37]. Compared with people who are dissatisfied with their jobs, those who believe that they have meaningful jobs that translate effectively into job satisfaction are likely to have better mental health through greater life satisfaction [84].

In addition to examining the independent mediating role of subjective well-being and life satisfaction, we also confirmed the multiple mediating effects of subjective well-being and life satisfaction on the relationship between job satisfaction and depression. The results of the present study demonstrate that higher job satisfaction is a possible promoter for subjective well-being and life satisfaction, which are, in turn, related to the decrease in depressive symptoms. Positive experiences and emotions in the work conditions can influence other areas of one’s life and bring about the same positive and pleasant feelings, namely, higher levels of subjective well-being [85]. At the same time, higher subjective well-being results in better life satisfaction. Subjective well-being and life satisfaction, which represent positive emotions, have not only been considered as protective factors against the adverse effects of negative emotions, but also as promoting factors of mental health [86]. Obtaining satisfying and meaningful jobs is important for Chinese adults aged 35–60 years in establishing overall well-being, which is also linked to greater life satisfaction, positive affect, and life meaning as well as lower depression [85,87].

### 4.3. Limitations

The results from the current study should be considered with some limitations. The study was cross-sectional, so we cannot determine causality nor can we speak to the directionality of the relation between job satisfaction and depression. As discussed above, higher job satisfaction can improve mental health, which may be helpful to decrease the risk of depression among Chinese adults aged 35–60 years. However, those with lower symptoms of depression may be more likely to perceive their job as satisfactory. Longitudinal and experimental studies are needed to determine the direction of this relation.

## 5. Conclusions

Depression has become one of the significant public health problems that seriously affects the physical and mental health of human beings. The combination of good mental health and freedom from depression is fundamental to our collective and individual ability as humans to think, emote, interact with each other, earn a living, and enjoy life [88]. The findings of our study indicate that both subjective well-being and life satisfaction are significant independent mediators of the relationship between job satisfaction and depression among Chinese adults aged 35–60 years, and also provide evidence that increasing job satisfaction could decrease depressive symptoms through promoting subjective well-being and life satisfaction. Our study has important reference significance in the continuing research in improving the mental health and reducing the risk of depression targeting adults aged 35–60 years in China.

## Figures and Tables

**Table 1:** Characteristics of the study participants (N = 10,609).

Variables	Category	N	Mean ± SD /Percentage (%)
Age			47.91 ± 6.88
Gender	Female	5202	49.0
	Male	5407	51.0
Self-reported health	Very unhealthy	1700	16.0
	Unhealthy	1590	15.0
	General	4567	43.0
	Healthy	1351	12.7
	Very healthy	1401	13.2
Marital status	Not married	531	5.0
	Married	10,078	95.0

Note: SD means the standard deviation.

**Table 2:** Correlations between job satisfaction, subjective well-being, life satisfaction, and depressive symptoms.

Variables	Job Satisfaction	Subjective Well-Being	Life Satisfaction	Depressive Symptoms
Job satisfaction	1			
Subjective well-being	0.144 ***	1		
Life satisfaction	0.228 ***	0.398 ***	1	
Depressive symptoms	−0.109 ***	−0.321 ***	−0.230 ***	1

Note: *** *p* < 0.001 (two-tailed).

**Table 3:** The association between job satisfaction and depressive symptoms.

Variables	Depressive Symptoms					
	Model 1			Model 2		
	B	t	*p*	B	t	*p*
Job satisfaction	−0.134	−16.113	<0.001	−0.0902	−11.3296	<0.001
Age				−0.0117	−2.2350	0.0254
Gender						
Female				1		
Male				−0.8347	−11.5946	<0.001
Self-reported health				0.9671	31.4952	<0.001
Marital status						
Not married				1		
Married				−1.7920	−10.9014	<0.001

**Table 4:** Hypothesized serial mediation model of job satisfaction, subjective well-being, life satisfaction, and depressive symptoms.

Pathway	Effect	SE	BootLLCI	BootULCI
Total effect (c)	−0.0902	0.008	−0.1058	−0.0746
Direct effect (c’)	−0.0371	0.0083	−0.0531	−0.0206
a1	0.0306	0.0022	0.0264	0.0348
a2	0.0366	0.0021	0.0327	0.0407
a3	0.3698	0.0108	0.3483	0.3905
b1	−1.0451	0.0434	−1.1301	−0.9607
b2	−0.4414	0.0438	−0.5292	−0.3578
Indirect effects				
Total indirect effects	−0.0531	0.0034	−0.0600	−0.0468
Indirect 1	−0.032	0.0026	−0.0373	−0.0270
Indirect 2	−0.0162	0.0019	−0.0200	−0.0126
Indirect 3	−0.005	0.0006	−0.0063	−0.0038

Note: Indirect 1, Job satisfaction→subjective well-being→depressive symptoms; Indirect 2, Job satisfaction→life satisfaction→depressive symptoms; Indirect 3, Job satisfaction→subjective well-being→ life satisfaction→depressive symptoms; BootLLCI = bootstrapping lower limit confidence interval; BootULCI = bootstrapping upper limit confidence interval; SE means standard error; Effect means standardized regression coefficient.

## Data Availability

The data can be obtained at: https://opendata.pku.edu.cn/dataverse/CFPS (accessed on 1 March 2022). Researchers are required to apply for permission to use the data.
